# Real-Time Remote Monitoring of Environmental Conditions and Actuator Status in Smart Greenhouses Using a Smartphone Application

**DOI:** 10.3390/s26051548

**Published:** 2026-03-01

**Authors:** Emmanuel Bicamumakuba, Md Nasim Reza, Hongbin Jin, Samuzzaman, Hyeunseok Choi, Sun-Ok Chung

**Affiliations:** 1 Department of Agricultural Machinery Engineering, Graduate School, Chungnam National University, Daejeon 34134, Republic of Korea; ebicamu@o.cnu.ac.kr (E.B.); reza5575@cnu.ac.kr (M.N.R.); samsuzzaman@o.cnu.ac.kr (S.); 2 Department of Smart Agricultural Systems, Graduate School, Chungnam National University, Daejeon 34134, Republic of Korea; jhb0117@o.cnu.ac.kr; 3 Korea Institute of Industrial Technology, Cheonan 31056, Republic of Korea; hchoi@kitech.re.kr

**Keywords:** smart greenhouse, smartphone application, wireless network, real-time monitoring, abnormalities detection

## Abstract

Advancement of precision agriculture increasingly relies on cost-effective and scalable technologies for real-time environmental management, particularly in greenhouse environments where vertical and spatial microclimate heterogeneity influences crop performance. This study presents the design, implementation, and experimental validation of an Android-based smartphone application edge supervisory monitoring system integrated with multi-layer wireless sensing and control nodes for real-time monitoring in a smart greenhouse. The system combined multi-layer wireless sensor nodes, wireless control nodes, a Long-Range Wide Area Network (LoRaWAN) gateway, Message Queuing Telemetry Transport (MQTT) communication, and a cloud-synchronized smartphone-based supervisory interface for visualizing environmental data, detecting defined abnormal events, and controlling actuators remotely. For feasibility tests, 54 sensing nodes and 12 actuator nodes were deployed across three vertical layers in two sections, measuring temperature, humidity, CO_2_ concentration, and light intensity. Abnormality was defined as environmental threshold violations, statistical signal deviations, actuator power inconsistencies, and communication timeout events. Experimental results revealed vertical and spatial environmental variability across greenhouse sections, while real-time time-series and 3D spatial maps enabled the rapid detection of abnormal conditions. The rule-based abnormality detection engine identified out-of-range environmental values and sensor-related inconsistencies and generated immediate notifications. Smartphone profiling revealed that display and system-level processes accounted for energy consumption, with battery power reaching a peak of 3.5 W and application CPU utilization ranging from 40% to 70% during active monitoring. The results demonstrate system-level feasibility, responsiveness, and scalability under commercial greenhouse workloads, supporting future integration of predictive control and energy-efficient operation.

## 1. Introduction

Smart greenhouses represent a significant technological advancement in protected cultivation, enabling growers to maintain crop growth within narrow environmental ranges while minimizing labor and resource waste [1]. Unlike conventional greenhouses, which rely heavily on manual inspections and on-site control actions, smart greenhouses integrate distributed sensing, automated actuation, and decision-support functions to regulate temperature, humidity, CO_2_ concentration, and light in real-time [2]. When environment control is handled manually, decisions are often delayed, inconsistent, and based on limited local observations, resulting in suboptimal conditions, yield loss, and inefficient use of energy and water [3]. In contrast, data-driven environmental management allows operators to maintain microclimate variables closer to crop-specific targets, reducing stress and improving yield quality and stability under increasingly variable weather and market conditions [4,5].

Over the past decades, the rapid development of Internet of Things (IoT) technologies has made it possible to deploy dense wireless sensor node networks in smart greenhouses. These networks measure temperature, relative humidity, CO_2_ concentration, light intensity, soil water, and other environmental variables at multiple locations and heights, enabling a high-resolution representation of smart greenhouse microclimate dynamics [6,7]. When combined with cloud platforms and edge computing, wireless node networks support continuous data logging, remote visualization, abnormality detection, and model-based environmental control [8]. Several recent reviews emphasized that IoT-enabled architecture has become the backbone of intelligent greenhouse systems, highlighting a transition from single-point sensing to distributed and multi-parameter environmental monitoring [9].

In parallel, advances in communication technologies, ranging from ZigBee to Wi-Fi, cellular networks, and especially low-power wide-area networks (LPWANs), have strengthened the connectivity of smart greenhouse monitoring systems [10,11]. LPWAN solutions such as LoRaWAN provide long-range, low-power communication in sub-GHz bands and have demonstrated stable performance for environmental sensing in protected cultivation systems [12]. Their ability to operate with low packet loss and extended battery life makes them suitable for large-scale, multi-node deployments in humid and electro-magnetically challenging smart greenhouse environments [13,14]. However, despite these advances, many studies still focus on basic monitoring or single-zone control and rarely address three-dimensional environmental variability in greenhouses [15,16].

The smartphone has emerged as one of the most practical human–machine interfaces for smart greenhouse applications. It offers strong processing capability, high-quality dis-plays, and constant connectivity while being widely available to growers [17]. Several prototypes and commercial systems have demonstrated that Android-based smartphone applications can visualize real-time environmental data, provide Android historical trends, and support basic actuator commands, such as fan activation, vent adjustment, or irrigation scheduling [18,19]. Nonetheless, many existing applications remain limited in scope or often connected to only a few sensors, lack advanced filtering or abnormalities detection algorithms, and do not incorporate spatial or vertical environmental mapping, which is crucial for understanding thermal and humidity gradients in large greenhouse zones [20]. In particular, the lack of mobile, spatially aware supervision tools limit growers’ ability to diagnose localized environmental issues during routine greenhouse operations. This study directly addresses these limitations by positioning the smartphone as a real-time supervisory interface that integrates sensing, visualization, anomaly detection, and actuator awareness within the greenhouse environment.

Despite these developments, practical gaps remain between research prototypes and the operational requirements of commercial smart greenhouses. Many existing monitoring platforms employ fixed dashboards or browser-based interfaces that, while functionally mobile capable, are less optimized for continuous in-field supervision and spatial diagnosis under dense, multi-node deployments [21]. Moreover, although communication latency and reliability have been extensively studied at the protocol level, system-level evaluations that integrate multi-layer sensing, actuator supervision, spatial visualization, and sustained mobile interaction under real greenhouse workloads remain limited. In addition, localized microclimate heterogeneity such as roof-level heat accumulation, canopy-level humidity pockets, and CO_2_ buildup has often been described qualitatively rather than quantitatively mapped during operation [22,23]. Accordingly, this study focuses on system-level integration and the quantitative validation of spatially resolved supervisory monitoring under commercial operating conditions, rather than on the development of new communication protocols or mobility frameworks [24]. Anomaly detection has been extensively investigated in IoT systems at physical, MAC, and routing layers; node-level spatial anomaly localization integrated with real-time mobile-edge visualization under commercial greenhouse deployment conditions remains comparatively limited [25].

Although smartphone-based greenhouse monitoring applications have been reported, most existing implementations are limited to basic time-series dashboards or simple actuator toggling interfaces connected to a small number of sensors [26]. These systems rarely incorporate vertically stratified sensing, spatial interpolation, integrated actuator diagnostics, and real-time abnormality evaluation within a unified mobile supervisory framework. Consequently, their ability to support high-resolution spatial supervision, node-level fault localization, and sustained multi-node traffic under commercial greenhouse workloads remains insufficiently validated [27].

In this study, an Android-based smartphone application was specifically designed and developed as an integrated supervisory monitoring platform capable of handling dense multi-layer environmental sensing and actuator data streams in real-time. The application was not developed merely as a visualization tool, but as a coordinated supervisory engine integrating (i) synchronized MQTT-based data streaming, (ii) three-dimensional spatial variability mapping using inverse distance weighting (IDW), (iii) deterministic rule-based abnormality detection executed at the mobile edge, (iv) actuator power monitoring with command-response verification, and (v) sustained performance profiling under continuous multi-node operation. The scientific contribution, therefore, lies in demonstrating that such integrated supervisory functionality remains computationally feasible, communication-stable, and operationally reliable within a commercial greenhouse deployment involving 54 sensing nodes and 12 actuator nodes over more than one million environmental observations.

To structure this validation, four measurable supervisory performance dimensions are defined: three-dimensional microclimate heterogeneity, spatial persistence and frequency of abnormal environmental and actuator events, end-to-end communication robustness under dense multi-node traffic, and smartphone computational feasibility assessed through battery, CPU, and memory profiling. These dimensions collectively evaluate supervisory effectiveness rather than representing independent research objectives.

The rest of this paper is organized as follows. Section 2 describes the materials and methods, including the architecture of the Android-based smartphone application, communication protocols, abnormality detection strategy, and the experimental setup in a commercial smart greenhouse. Section 3 presents the experimental results, focusing on vertical and spatial environmental variability, real-time smartphone visualization, abnormal-signal detection, and smartphone energy and performance evaluation. Section 4 discusses the main findings, system performance, and limitations in the context of existing smart greenhouse monitoring approaches. Section 5 concludes the paper and outlines directions for future work.

## 2. Materials and Methods

### 2.1. Smartphone Application Framework Within the LoRaWAN-Based Monitoring Architecture

**Figure 1:**
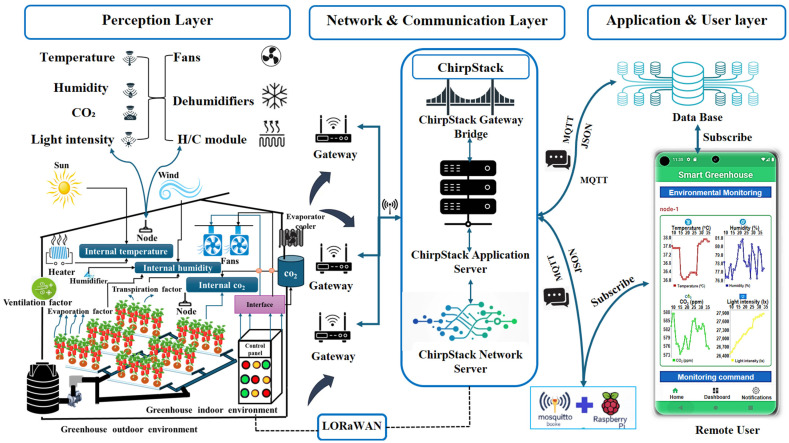
System architecture of the LoRaWAN-based smart greenhouse monitoring and control platform integrating multi-layer environmental sensor nodes, actuator nodes, ChirpStack network services, MQTT communication, cloud database, and an Android-based smartphone application.

The proposed smart greenhouse platform was implemented as a layered LoRaWAN-based architecture comprising a perception layer, a network and communication layer, and an application and user layer, as illustrated in Figure 1. Multi-layer environmental sensor nodes and actuator nodes constitute the perception layer, while LoRaWAN communication and ChirpStack network services provide reliable data transmission, device management, and application-level integration. Within this framework, the smartphone application functions as a mobile supervisory interface for real-time monitoring, anomaly inspection, and actuator supervision rather than as a primary control component.

The Android-based smartphone application was developed using Android Studio with Java to ensure broad device compatibility and long-term API stability [28]. Kotlin coroutines were employed to support asynchronous data reception, real-time visualization, and command handling without blocking the user interface [29]. Secure user access was enforced using Firebase authentication.

Environmental and actuator data acquired by distributed sensor nodes were transmitted through the LoRaWAN gateway and processed through the ChirpStack Gateway Bridge, Network Server, and Application Server. Data were then delivered to the application layer using the MQTT protocol, enabling low-latency, bidirectional communication between the cloud infrastructure and the smartphone application. A cloud database supports data persistence, synchronization, and historical access [30,31].

This architecture enables reliable end-to-end data flow and supports spatially resolved environmental monitoring in heterogeneous greenhouse environments. Detailed low-level application implementation details are omitted, as the focus of this study was on the integrated sensing communication visualization pipeline and its effectiveness for real-time supervisory greenhouse management.

#### 2.1.1. Android Software Development Tools Used

**Figure 2:**
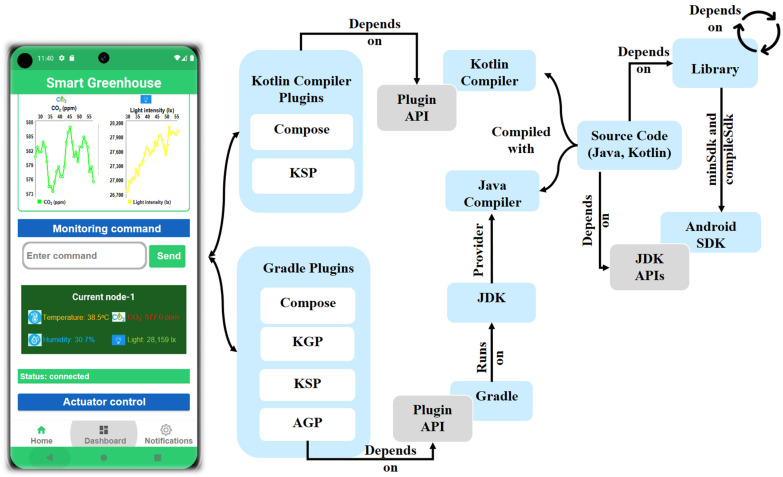
The software builds a tools framework and dependency relationships for the development of Android-based smartphone applications.

The software development tools used for implementing Android-based smartphone applications were structured to define clear relationships among the application code, external library dependencies, and build-time tools, as illustrated in Figure 2. The build process was managed using the Gradle build system, which orchestrates compilation, dependency resolution, and packing through standardized plugin interfaces.

The application source code was implemented primarily in Java with optional Kotlin support. Java sources were compiled using the Java Development Kit (JDK), while Kotlin sources were compiled using the Kotlin compiler integrated through the Android Studio and Gradle build toolchain. The compilation process was extended through compiler and build time plugins accessed via standardized plugin application programming interfaces (APIs). Kotlin-related tooling, including Jetpack Compose and Kotlin Symbol Processing (KSP), was used to enable advanced language features and build-time code generation when required [32].

Gradle plugins, including the Android Gradle plugin (AGP), Kotlin Gradle Plugin (KGP), and associated tooling, were installed and managed within the Gradle environment to configure build variants, handle resource processing, and enforce compatibility with the Android Software Development Kit (SDK). These plugins interact with the compiler toolchain through the plugin API, ensuring consistent integration between source code, libraries, and platform requirements [33].

External libraries were incorporated as build-time dependencies and resolved according to the minimum and target Android SDK versions. The Android SDK, together with its associated application programming interfaces (APIs), provided the platform-level abstractions required for application compilation and packaging [34]. This building framework ensures that application code, third-party libraries, and platform tools are coherently integrated, enabling reproducible builds, dependency traceability, and long-term maintainability of the Android-based smartphone application.

#### 2.1.2. Smartphone Application User Interface Architecture and Interaction Design

**Figure 3:**
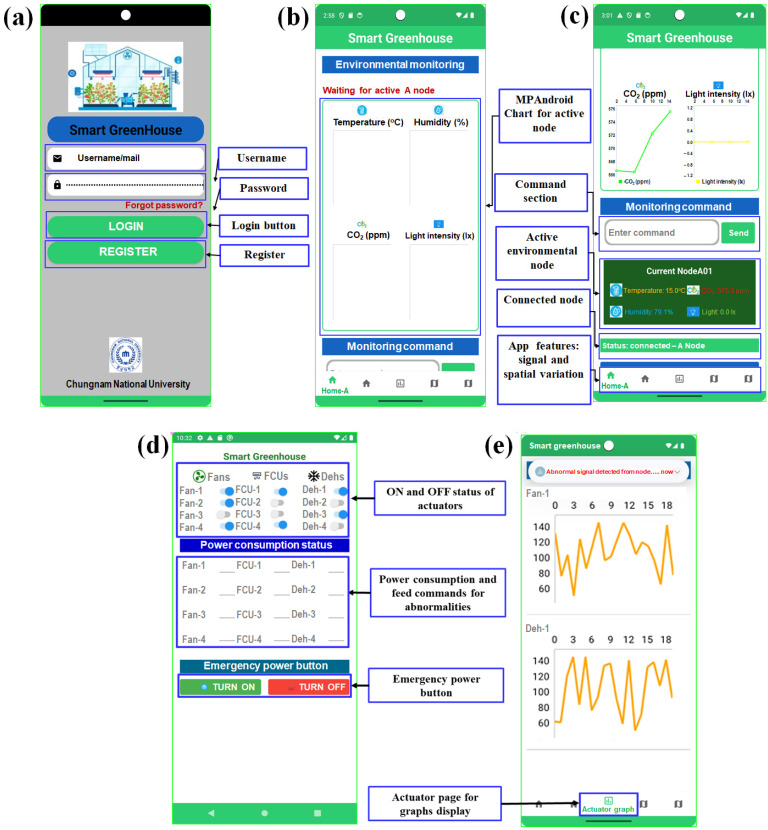
Screenshot of the graphical user interface (GUI) developed in Android Studio for (**a**) the login page, (**b**) environmental monitoring sensing data by MPAandroidChart, (**c**) the command section with other app features, (**d**) the actuator control page, and (**e**) the actuator page for graphical visualization.

The user interface (UI) was designed to provide an operationally robust, low-latency supervisory environment for smart greenhouse management. Developed in Android Studio using XML layouts and Jetpack Compose components, the UI adheres to material design principles to ensure clarity and usability in various lighting conditions [32]. As illustrated in Figure 3, the interface was organized into four functional screens. The login page for secure authentication module powered by Firebase authentication is shown in Figure 3a, a real-time monitoring dashboard incorporating MPAndroidChart for temperature (Figure 3b,c), humidity, carbon dioxide concentration, and light intensity visualization (Figure 3d), and an actuator control panel for fans, H/C module units, and dehumidifiers with integrated power consumption status and an emergency shutdown function; and anomaly detection displays presenting abnormal signal patterns through time series plots (Figure 3e).

Layout structure combined constraint layout and linear layout to maintain device-independent responsiveness, while touch elements such as switches and command buttons were optimized for precise interaction in operational environments. Real-time UI updates were driven through view model state observers and Kotlin coroutine flows, enabling the continuous rendering of sensor data and anomaly alerts without blocking the user interface. A dedicated configuration interface allows operators to input MQTT broker parameters and system settings through validation to prevent misconfiguration. The interface architecture provides a compact, responsive, and operator-centered interaction layer that integrates environmental monitoring, actuator supervision, anomaly inspection, and system configuration into a cohesive workflow suitable for continuous operation in a smart greenhouse.

#### 2.1.3. Spatial Variability Visualization Module for Environmental Parameters

A spatial variability visualization module was implemented using an embedded WebView component to render real-time temperature and humidity heatmaps within the smartphone application. The module leverages the dimensions of the smart greenhouse height (4.5 m), width (7.0 m), and length (27.0 m) to create a 3D spatial map that reflects the environmental conditions at various points in the structure. During application initialization, the layout for the heatmap view was loaded, JavaScript execution was enabled, and the WebView environment was configured to support interactive rendering. The WebView subsequently loaded a locally stored HTML resource containing JavaScript routines responsible for generating the spatial variability maps based on defined greenhouse geometry.

**Figure 4:**
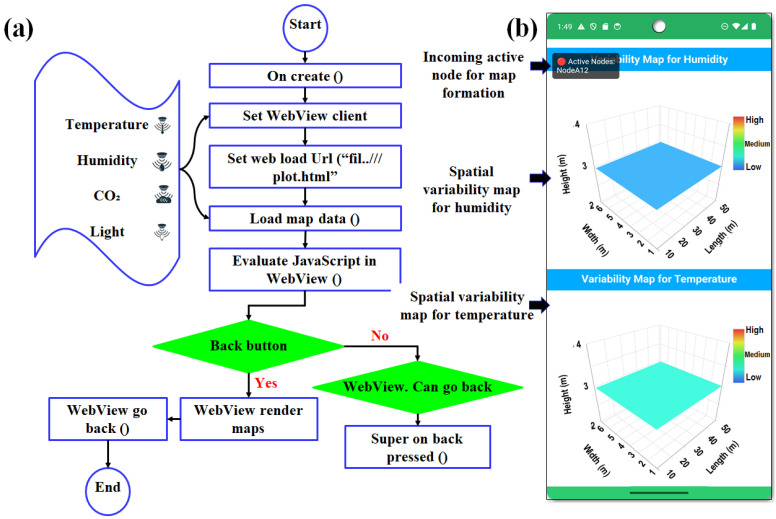
(**a**) Flowchart of the variability map developed in the smartphone application, and (**b**) screenshot of the smartphone application showing the variability map for temperature and humidity.

Environmental data received from sensor nodes via MQTT or Firebase were parsed into a structured node-based format and dynamically injected into WebView through JavaScript interface calls. These interactions updated the internal temperature and humidity data layers within the HTML rendering engine and triggered redraw operations, allowing the heatmap visualization to be refreshed continuously without reloading the entire page, as illustrated in Figure 4a,b. Spatial interpolation for three-dimensional visualization was performed using the inverse distance weighting (IDW) method. An IDW power parameter of *p* = 2 was selected to balance local influence and spatial smoothness, which is commonly applied in greenhouse environmental mapping. Interpolation was performed using all available sensor nodes within each greenhouse section, without restricting neighborhood size, to preserve spatial continuity across the monitored domain. Boundary handling was constrained to the physical greenhouse geometry (width, length, and height), and no extrapolation was performed beyond the sensor deployment limits. This configuration ensured that spatial maps accurately reflected the measured conditions while avoiding artificial edge artifacts. Design enabled the smooth and continuous visualization of spatial environmental variability, capturing temperature and humidity variability across the height, width, and length dimensions of the smart greenhouse.

Interactive navigation logic was incorporated to allow users to explore previous states of the heatmap visualization. The back navigation behavior was customized so that the interface returned to earlier visualization states when available, rather than immediately terminating the view. By integrating Android side layout management with JavaScript-based rendering within WebView, the spatial variability module provided an efficient, lightweight, and platform-independent visualization pipeline. This architecture supports the real-time analysis of smart greenhouse environmental conditions and offers operators a responsive tool for environmental interpretation and control.

#### 2.1.4. Real-Time Abnormality Detection and Notification Strategy

The abnormality detection module was fully implemented directly within the smartphone application as an integrated rule-based statistical evaluation engine. Each incoming sensor packet, delivered through MQTT or Firebase, was parsed on the device and evaluated locally to ensure immediate detection, without relying on cloud round-trip latency. In the context of this study, abnormality was defined as any deviation from agronomically acceptable environmental conditions, statistically expected sensor behavior, or actuator performance consistency at the node level. To ensure methodological clarity, abnormal events were classified into three primary categories: (i) environmental operational range violations relative to crop-specific thresholds, (ii) statistical transient deviations from rolling mean behavior within nominal ranges, and (iii) actuator behavioral inconsistencies including abnormal power consumption, command–response mismatch, or loss of heartbeat signal. This structured definition differentiates crop stress conditions from sensor instability, communication faults, and actuator malfunction within the supervisory monitoring framework. Thresholds were selected based on commonly reported greenhouse operational ranges for strawberry cultivation and may vary depending on cultivar and growth stage as shown in Table 1.

Where $x_{i,t}$ denotes the environmental measurement at the node i and time t; $x_{min}$ and $x_{max}$ represent crop-specific operational bounds; $\mu_{i,w}\;$ and $\sigma_{i,w}$ denote the rolling mean and standard deviation computed over a window of size w (15 sampling intervals in this study); $c_{i,t}$ represents the measured actuator power, and $c_{i}^{expected}$ is the nominal calibrated value; $\delta_{c}$ is the allowable power deviation ratio (0.20); $u_{i,t}$ indicates the actuator command state; τ denotes the elapsed time after actuator activation; and $T_{response}$ and $T_{timeout}$ represent the actuator response and communication timeout thresholds, respectively.

Environmental measurements collected from each sensor node were expressed as $x_{i,t}=\{T_{t},H_{t},C_{t},L_{t}\}$ where $T_{t},H_{t},C_{t},L_{t}$ represent temperature, relative humidity, carbon dioxide concentration, and light intensity at time t. Each parameter $x_{t}$ was evaluated using a threshold-based rule engine that compared the measurement user-defined limits $\left[x_{min},x_{max}\right]$. An abnormal condition was detected when the parameter exceeded this operational range defined by:

(1) $$Abnormality(x_{t})=\{\begin{array}{cc} 1, & x_{t}\notin [x_{min},x_{max}]\\ 0, & normal \end{array}$$

where 1 indicates an abnormal operation, and 0 indicates normal operation. To detect transient fluctuations within nominal limits, the smartphone application maintains rolling windows $W_{p}$ for each parameter p∈{T,H,C,L} and computes the mean $\mu_{p}$ and standard deviation $\sigma_{p}$. A statistical anomaly was triggered when the current measurement deviated beyond three standard deviations:

(2) $$|p_{t}-\mu_{p}|\;>3\sigma_{p}$$

where $p_{t}$ is the most recent value, $\mu_{p}$ is the rolling mean, and $\sigma_{p}$ is the rolling standard deviation for the parameter p. Actuator diagnostics were implemented in parallel, where missing responses, abnormal power deviations, $|c_{t}-c_{\mathrm{expected}}|\;>\delta_{c}$, or ineffective operation trends trigger actuator-fault events. $c_{t}$ is the instantaneous power reading and $\delta_{c}$ is the permissible deviation margin. Ineffective operation, such as active dehumidification with no corresponding change in humidity, was also categorized as a fault. When any abnormality was detected, the smartphone application generated a structured event:

(3) $$E=\;\{\;i,t,x_{t},\mathrm{type},\;\mathrm{severity}\}$$

where i is the node identifier, t is the timestamp, $x_{t}$ represents the associated sensor readings, and “type” and “severity” denote the anomaly classification.

**Figure 5:**
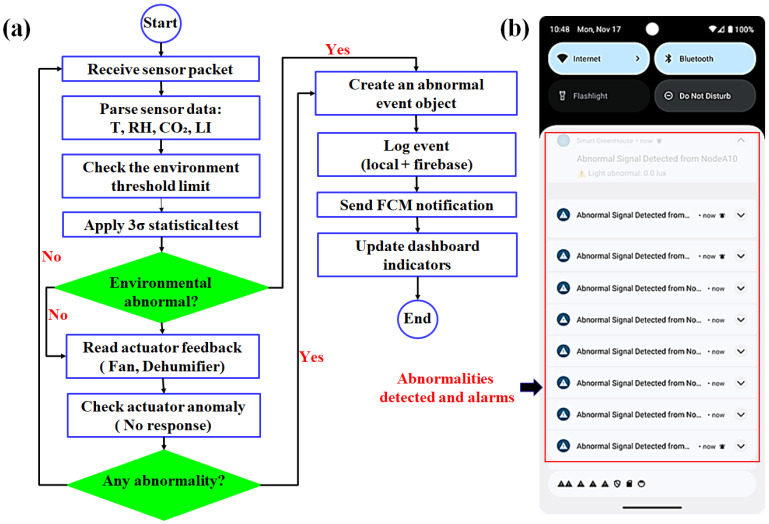
(**a**) Flowchart of the abnormal detection and notification process in the smartphone application, and (**b**) screenshot of abnormal detection and notification alarms in a smartphone application.

The event was logged locally and synchronized to Firebase for historical reference. Push notifications are dispatched through Firebase Cloud Messaging (FCM), ensuring timely alerts even during background operation, while the dashboard provides color-coded visual cues for rapid interpretation. Alert grouping minimizes notification fatigue and supports efficient decision-making. The complete detection workflow and alert interface are shown in Figure 5. 

#### 2.1.5. Smartphone Application Cloud Interface for Data Communication Protocol

Environmental and actuator data were transmitted from sensing and actuator nodes using the LoRaWAN protocol and forwarded through an edge device to the cloud using the MQTT publish-subscribe communication model. LoRaWAN enables low-power, long-range uplink transmission from distributed nodes to the gateway, while MQTT ensures reliable and lightweight data delivery between the edge layer and cloud server. MQTT quality of service (QoS) level 1 was applied to guarantee message delivery with acknowledgment.

Sensor and actuator data were encapsulated in a lightweight JSON-based key-value format containing time-stamped environmental variables (temperature, humidity, CO_2_ concentration, light intensity) and actuator parameters (power consumption). This structured format supports efficient parsing, low communication overhead, and compatibility with cloud databases and smartphone applications. The payload size of a single message is defined as:

(4) $$S_{\mathrm{payload}}=\sum_{r=1}^{n}\left(B_{k,i}+B_{v,i}+B_{t}\right)$$

where N is the number of transmitted fields, $B_{k,i}$ and $B_{v,i}$ denote the byte lengths of the key and value of the i-th field, and $B_{t}$ represents the timestamp size. For a sampling interval $T_{s}$, the data transmission rate per node $R_{d}$ is expressed as:

(5) $$R_{d}=\frac{S_{\mathrm{payload}}}{T_{s}}$$

**Figure 6:**
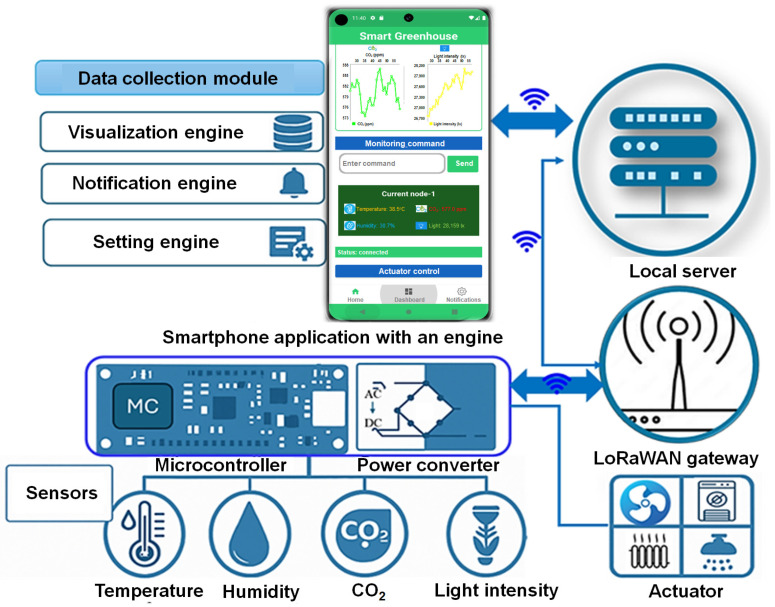
Data communication protocol from environmental sensing and actuator to the smartphone application engine.

After reception at the gateway, MQTT messages were processed at the edge layer using the Python (Ver. 3.12)-based scripts, temporarily buffered during network interruptions, and uploaded to the cloud database for real-time synchronization with the smartphone application. This protocol format combination ensures secure, low-latency, and scalable data transmission suitable for continuous smart greenhouse monitoring, as illustrated in Figure 6. 

#### 2.1.6. Environmental Sensing Nodes and Hardware Configuration

The environmental sensing nodes were designed to monitor key microclimate variables relevant to smart greenhouse operation, including air temperature (T), relative humidity (RH), CO_2_ concentration, and light intensity (LI). Each node integrated calibrated, low-power sensing elements selected to ensure measurement reliability under high-humidity and temperature-variable greenhouse conditions.

Air temperature and relative humidity were measured using a digital temperature-humidity sensor (DHT22; Aosong Electronics Co Ltd., Guangzhou, China), providing a measurement range of −40 to 80 °C and 0 to 100% RH, with accuracies of ±0.5 °C and ±2%, respectively. These specifications support the reliable detection of vertical and spatial microclimate variation within the greenhouse canopy and upper layers. CO_2_ concentration was measured using a nondispersive infrared (NDIR) sensor (MH-Z19B; Zhengzhou Winsen Electronics Technology Co., Ltd., Zhengzhou, China) operating over a 0 to 5000 ppm range with ±50 ppm accuracy, enabling the assessment of ventilation effectiveness and localized CO_2_ accumulation. Light intensity was monitored using a photodiode-based digital sensor (BH1750; Shenzhen Youxin Electronic Technology Co., Ltd., Guangdong, China) with a detection range of 1 to 65,535 lx, suitable for capturing diurnal solar radiation patterns and shading effects.

All sensors were selected for low power consumption, long-term stability, and compatibility with periodic calibration. Factory calibration was verified prior to deployment using reference instruments under controlled conditions, and routine zero-offset and span checks were performed during maintenance to ensure data consistency throughout the monitoring period. The specifications and model numbers of the environmental sensors used in this study are summarized in Table 2.

#### 2.1.7. Power Monitoring Nodes and Hardware Configuration

Power monitoring nodes were implemented to ensure electrical safety and enable the real-time monitoring of actuator energy consumption in the smart greenhouse. These nodes measured the operating current of AC and BLDC fans, as well as AC and BLDC dehumidifiers, allowing the detection of abnormal power usage and actuator malfunction. Current measurements were obtained using a Hall-effect current sensor module (ACS712-30A; Allegro MicroSystems, Inc., Manchester, NH, USA), providing a bidirectional measurement range of ±30 A with ±1.5% accuracy. The sensor measured load current via the IP+ and IP− terminals and generated an analog voltage proportional to the instantaneous current. This signal was sampled by the analog-to-digital converter of the Arduino MKR WAN 1310 microcontroller, enabling real-time estimation of actuator power consumption.

**Figure 7:**
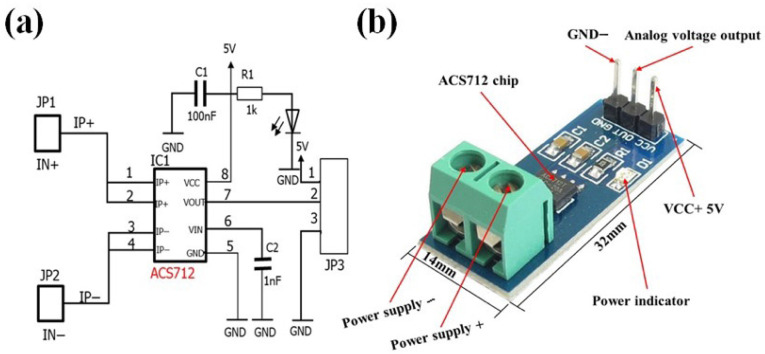
ACS712 Hall-effect current sensor module: (**a**) circuit schematic of the current sensing configuration; (**b**) physical module showing the IP+/IP− terminals, analog output, and power connections.

The power monitoring circuit was supplied via a regulated 5 V USB input. Decoupling and smoothing capacitors were incorporated at the sensor output and supply terminals to stabilize the voltage and reduce electrical noise, which is critical in electrically dynamic greenhouse environments with frequent actuator switching. This configuration supports reliable overload detection, abnormal operation identification, and energy usage assessment. The specifications of the actuator power monitoring sensor are summarized in Table 3. A detailed circuit diagram illustrating the ACS712 current sensor module, power supply connections, signal conditioning components, and microcontroller interface is shown in Figure 7.

#### 2.1.8. LoRaWAN Communication Configuration and Security

The wireless communication layer of the proposed smart greenhouse monitoring platform was implemented using the LoRaWAN protocol to ensure energy-efficient, long-range, and secure data transmission from distributed sensor nodes to the central server. All environmental and power monitoring nodes operated in the 923 MHz ISM band and were configured as Class A LoRaWAN end devices, enabling ultra-low-power operation through uplink-initiated communication and scheduled downlink reception windows.

Uplink transmissions employed a 125 kHz channel bandwidth with adaptive spreading factors (SF7-SF10) to accommodate spatial variability in link quality across the greenhouse. Adaptive data rate (ADR) was enabled to dynamically optimize spreading factor and transmission parameters based on network conditions, thereby reducing airtime, minimizing packet collisions, and extending battery life while maintaining reliable communication. The transmission output power was set to 14 dBm, consistent with regional regulatory constraints and greenhouse deployment requirements.

Network access and device authentication were performed using over-the-air activation (OTAA). Each sensor node was provisioned with a unique Application Key (AppKey), allowing secure derivation of the Network Session Key (NwkSKey) and Application Session Key (AppSKey) during the join procedure. This mechanism ensured end-to-end encryption, data integrity, and protection against unauthorized access at both the network and application layers.

**Figure 8:**
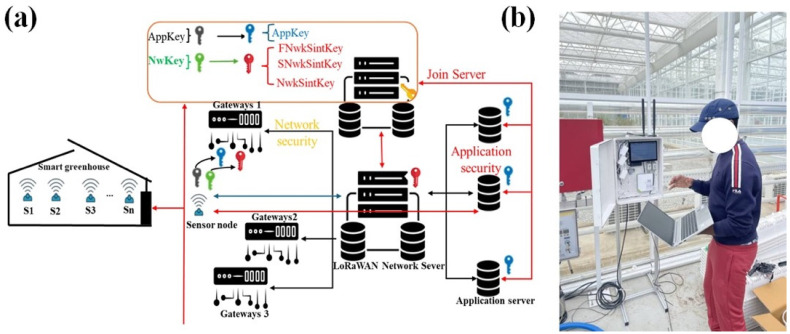
(**a**) LoRaWAN communication architecture and security framework illustrating OTAA authentication, key management (AppKey, NwkSKey), and encrypted data transmission between sensor nodes, gateway, and application servers, and (**b**) experimental deployment of the LoRaWAN gateway and edge device in the smart greenhouse environment for real-time data transmission to the smartphone application.

A Raspberry Pi 4 Model B equipped with a SenseCAP LoRaWAN concentrator functioned as the gateway, receiving uplink packets from multiple sensor nodes and forwarding them to the network server using a packet–forwarder interface. Received data were transmitted to the cloud server using the MQTT protocol with Quality of Service (QoS) level 1, guaranteeing reliable message delivery while maintaining low latency suitable for real-time smartphone visualization and control. To improve robustness under intermittent network conditions, sensor data were temporarily buffered at the edge device before synchronization with the cloud database. The complete LoRaWAN communication architecture, security framework, and experimental deployment are illustrated in Figure 8.

#### 2.1.9. Feedback Command and Fault Recovery Mechanism

A feedback command and fault recovery mechanism was implemented to enhance control reliability and enable autonomous recovery from system malfunctions in the smart greenhouse. As illustrated in Figure 7, the mechanism establishes a bidirectional feedback loop among sensor nodes, the edge controller, and the smartphone application, comprising feedback commands and feedback messages. Each sensor node sends a periodic heartbeat message to indicate normal operation. $T_{h}$ is the alarm interval, and $t_{k}$ is the reception time of the *k*-th alarm. The elapsed time since the last alarm was defined as:

(6) $$\Delta t=t_{\mathrm{current}}-t_{k}$$

A malfunction condition M is detected when the elapsed time exceeds a predefined timeout threshold $T_{to}$.

(7) $$M=\{\begin{array}{cc} 1, & \Delta t>T_{to}\\ 0, & \Delta t\leq T_{to} \end{array}$$

where $T_{to}=10\;s$. Once a malfunction was detected, the fault type was encoded using a hexadecimal command code $C_{\mathrm{hex}}$, which represents what went wrong (sensor not responding, out-of-range data, frozen readings, actuator failure). A binary command code defines the corresponding recovery action $C_{\mathrm{bin}}$, which specifies what action to execute, such as restart, recalibration, status reporting, or forced shutdown. The recovery command selection can be expressed as:

(8) $$C_{\mathrm{bin}}=f(C_{\mathrm{hex}})$$

where $f(C_{\mathrm{hex}})$ denotes a deterministic fault-to-action mapping function and $C_{\mathrm{hex}}$ represents the hexadecimal-encoded fault identifier. The function f(⋅) maps each detected fault code to the corresponding binary recovery command $C_{\mathrm{bin}}$, defining the recovery action to be executed. The fault occurrence rate $R_{m}$ over an observation period $T_{o}$ is defined as:

(9) $$R_{m}=\frac{N_{m}}{T_{o}}$$

where $N_{m}$ is the number of detected malfunction events. The automatic recovery success ratio $\eta_{r}$ is expressed as:

(10) $$\eta_{r}=\frac{N_{r}}{N_{m}}$$

where $N_{r}$ is the number of malfunctions successfully resolved through automatic recovery commands.

**Figure 9:**
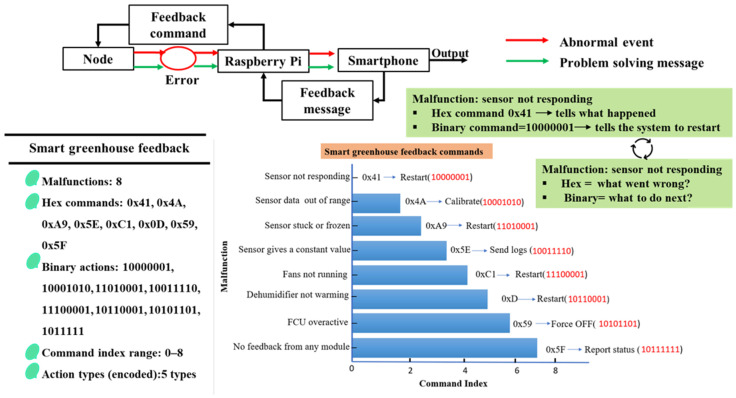
The feedback command structure illustrates the communication between the smartphone application and the nodes.

Upon issuing a recovery command, the edge controller generates a feedback message that is transmitted to the smartphone application, providing real-time notifications and diagnostic information. This enables users to monitor fault conditions and recovery outcomes while allowing the system to operate remotely. By combining alarm-based monitoring, deterministic fault detection, structured hexadecimal and binary command codes, and automatic recovery actions, the proposed feedback mechanism improves system robustness, reduces downtime, and ensures stable smart greenhouse operation. Figure 9 summarizes the feedback command structure, malfunction categories, and corresponding recovery actions implemented in the system. Recovery commands are limited to logical restart and communication recovery functions. Physical sensor recalibration was performed manually during maintenance and was not executed autonomously through software commands.

### 2.2. Experimental Site and Node Deployment in a Commercial Greenhouse

The experiment was conducted in a commercial smart greenhouse located in Goheung, Jeollanam-do, Republic of Korea (34.6° N, 127.3° E), a coastal agricultural region with a mild temperate climate. The greenhouse was selected due to its commercial-scale operation and suitability for evaluating real-time environmental monitoring and actuator behavior under practical operating conditions. The structure was a single-span arch-type greenhouse constructed with a steel frame and transparent plastic covering, commonly used in protected horticulture in Korea. The greenhouse was cultivated with strawberries throughout the experimental period, and all environmental interpretation (transpiration behavior, canopy structure, CO_2_ dynamics, and humidity patterns) was conducted under strawberry crop conditions. The real-time remote monitoring was conducted continuously over two weeks in late autumn at a fixed sampling interval of 1 min, following node-level functional verification and timestamp synchronization. With 54 environmental sensing nodes deployed, this protocol yielded approximately 20,160 time-stamped measurements per sensor and more than 1.08 million quality-controlled environmental data points in total, excluding actuator status and power monitoring records.

#### 2.2.1. Greenhouse Layout

A smart greenhouse was divided into three distinct study areas, designed as Area A, Area B, and Area C, to support experimental zoning and comparative spatial evaluation of the environmental conditions. Area A and Area C were selected as primary monitoring zones and were equipped with environmental sensing and actuator nodes to capture spatial and vertical variations in smart greenhouse conditions. These two areas represent actively monitored sections where real-time data collection and control were implemented.

Area B was intentionally excluded from sensing node deployment. This area served as a non-instrumented zone due to the structural and operational constraints and was maintained without sensor installation to avoid interference with experimental measurements. The absence of sensing nodes in Area B also enabled a comparative assessment between monitored and non-monitored zones within the same smart greenhouse environment.

**Figure 10:**
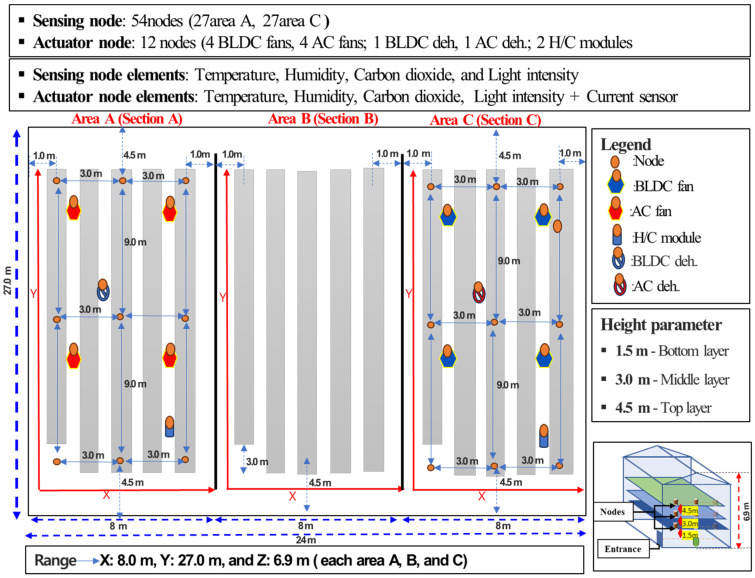
Schematic layout of environmental sensors and actuator deployment in a commercial greenhouse.

The zoning strategy enabled a systematic comparison of environmental conditions between the instrumented areas (Area A and Area C) and the non-instrumented reference area (Area B), providing spatial coverage for evaluating environmental heterogeneity and the effectiveness of sensor-based monitoring and control, as illustrated in Figure 10.

#### 2.2.2. Node Deployment and System Integration

A total of 54 environmental sensing nodes and 12 actuator nodes were deployed across Area A and Area C to enable coordinated monitoring and control of smart greenhouse conditions. Each area was equipped with 27 sensing nodes, distributed to provide balanced spatial coverage and capture environmental variability within the monitored zones. The sensing nodes measured temperature, relative humidity, CO_2_ concentration, light intensity, and electrical current, forming the primary data source for environmental assessment.

To capture vertical microclimate characteristics, sensing nodes were arranged at three height levels: 1.5 m of the bottom layer to represent plant environment interactions, 3.0 m of the middle layer to observe vertical airflow behavior and support sensor consistency, and 4.5 m of the upper layer to assess heat accumulation near the roof. This vertical stratification supports detailed spatial analysis and underpins subsequent variability mapping and anomaly detection.

**Figure 11:**
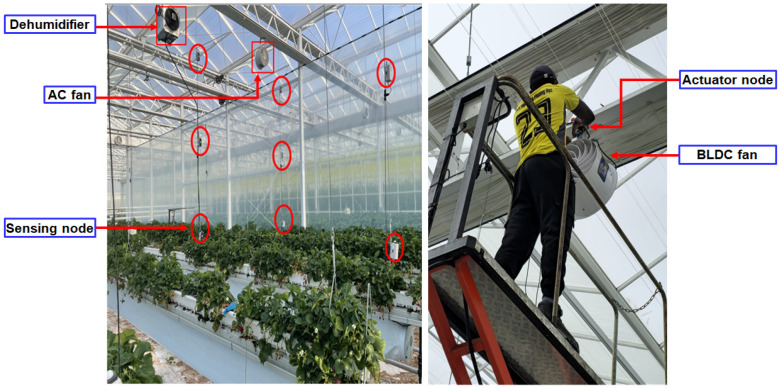
Environmental sensing and actuator node deployment and system integration in a commercial greenhouse.

In parallel, 12 actuator nodes were deployed in Area A and Area C to regulate environmental conditions based on real-time sensor feedback. These actuator nodes control the AC and BLDC fans, AC and BLDC dehumidifiers, and heating and cooling modules, enabling active adjustment of temperature, humidity, and airflow, as illustrated in Figure 11. Environmental sensing and actuator nodes were integrated within a unified control framework, allowing sensor-derived information to directly drive control actions. This coordinated deployment supports real-time automation, stable operation, and closed-loop environmental regulation throughout the smart greenhouse. 

### 2.3. Experimental and Analytical Procedures

The experimental procedures validated the Android-based smartphone application as the primary supervisory interface under commercial smart greenhouse conditions for strawberry cultivation. Validation focused on four criteria: (i) real-time data delivery, (ii) stability of continuous user interface (UI) rendering and spatial visualization, (iii) reliability of remote actuator command execution, and (iv) on-device anomaly detection and alerting. During monitoring, the application maintained a persistent MQTT subscription and Firebase synchronization while rendering time-series charts and spatial heatmaps. Incoming messages were time-stamped at receipt, parsed locally, evaluated by the abnormality detection engine, and rendered to the UI layer.

Application feasibility under multi-node traffic was evaluated using Android Studio Profiler by recording battery power consumption, CPU utilization, and memory usage throughout the monitoring period [35,36,37]. Validation metrics included uninterrupted message reception, sustained UI rendering without blocking, successful command response confirmation for actuators, and correct triggering of anomaly alerts. Smartphone feasibility indicators were quantified using average battery power, total energy consumption, mean CPU utilization, and net memory growth, defined using standard mobile and IoT profiling metrics [38,39,40].

Battery demand was evaluated using average battery power and total energy consumption, computational overhead using the mean CPU utilization attributed to the application, and memory stability using net memory growth over time, defined as:

(11) $${\bar{P}}_{\mathrm{bat}}=\frac{1}{T_{\mathrm{obs}}}\int_{0}^{T_{\mathrm{obs}}}P_{\mathrm{bat}}(t)\;dt$$

where ${\bar{P}}_{\mathrm{bat}}$ is the average battery power during monitoring (W), $P_{\mathrm{bat}}\left(t\right)$ is the instantaneous battery power at time t, and $T_{\mathrm{obs}}$ is the profiling duration (s).

(12) $$E_{\mathrm{obs}}=\int_{0}^{T_{\mathrm{obs}}}P_{\mathrm{bat}}(t)\;dt$$

where $E_{\mathrm{obs}}$ is the total energy consumed during monitoring (J), $P_{\mathrm{bat}}\left(t\right)$ is the instantaneous battery power (W), and $T_{\mathrm{obs}}$ is the observation period (s).

(13) $${\bar{U}}_{\mathrm{CPU},\mathrm{app}}=\frac{1}{T_{\mathrm{obs}}}\int_{0}^{T_{\mathrm{obs}}}U_{\mathrm{CPU},\mathrm{app}}(t)\;dt$$

where ${\bar{U}}_{\mathrm{CPU},\mathrm{app}}$ is the mean CPU utilization attributed to the application (%), $U_{\mathrm{CPU},\mathrm{app}}\left(t\right)$ is the instantaneous application of CPU utilization at time t, and $T_{\mathrm{obs}}$ is the profiling duration.

(14) $$\Delta M_{\mathrm{app}}=M_{\mathrm{app}}(T_{\mathrm{obs}})-M_{\mathrm{app}}(0)$$

where $\Delta M_{\mathrm{app}}$ is the net memory growth over the monitoring session (MB), *M_app_*(0) is the application memory footprint at session start, and *M_app_(T_obs_)* is the memory footprint at the end of the session.

To ensure that the study objectives were measurable, the evaluation focused on quantifiable system outputs rather than qualitative demonstration. Microclimate heterogeneity was quantified using node-level statistics aggregated by section and layer (Table 3) and through spatial interpolation maps. Abnormality detection performance was quantified using event frequency and affected-node distributions (Table 4), supported by visual inspection of representative alarms. Smartphone feasibility was quantified using profiling-derived battery power, CPU utilization, and memory footprint over time. Together, these metrics define the measurable performance envelope of the proposed supervisory monitoring approach under commercial deployment conditions.

Environmental parameters were evaluated against crop-based operational ranges covering both daytime and nighttime conditions. For strawberry cultivation, air temperature was evaluated within 5–40 °C, humidity within 40–90% [41], CO_2_ concentration within 350–1500 ppm, and light intensity within 0–15,000 lx, where 0 lx represents nighttime conditions [42]. Measurements exceeding these bounds were classified as abnormal. Actuator behavior was evaluated using the expected response characteristics and power deviation thresholds, with abnormal conditions identified when responses were missing, power consumption deviated by more than ±20%, or actuator activation failed to induce corresponding environmental changes. These criteria formed the basis for the anomaly detection and performance evaluation reported in Section 3.

## 3. Results

### 3.1. Vertical Variability of Environmental Conditions

During the experimental period, vertical variability was observed in temperature, humidity, CO_2_ concentration, and light intensity across both Section A and Section C, with consistent differences among the bottom layers (01–09 nodes), middle layers (10–18 nodes), and top layers (19–27 nodes). Temperature exhibited daytime stratification, with the top layer reaching the highest values, followed by the middle layers. Peak daytime temperature approached 30 °C in Section A and exceeded 35 °C in Section C, while nighttime temperatures became more uniform across layers, indicating reduced vertical variability under lower solar input. Relative humidity showed an inverse vertical pattern to temperature, with high values at the bottom layer and lower values at the top layer, particularly during daytime. At night, the humidity levels increased and became more homogeneous across all layers in both areas.

CO_2_ concentration displayed distinct vertical and spatial characteristics. In the section, relatively stable diurnal oscillations were observed with concentration generally higher at the bottom and middle layers than at the top layer. In contrast, Section C exhibited pronounced episodic CO_2_ accumulation at the bottom layer, with concentration temporally exceeding 1500 ppm, suggesting localized buildup and limited vertical mixing during specific periods. Light intensity demonstrated the biggest vertical difference among all variables, with maximum values consistently recorded at the top layer, followed by the middle and bottom layers. A clear day–night cycle was evident, and light intensity decreased to zero during nighttime, confirming the absence of artificial lighting. To quantitatively support the visually observed vertical and temporal variability, node-level descriptive statistics were computed for each environmental parameter over the two-week monitoring period. Table 4 summarizes the mean of node means, the standard deviation across nodes (spatial variability), and the median interquartile range (Q3–Q1) for Sections A and C, providing statistical depth beyond time-series visualization.

**Figure 12:**
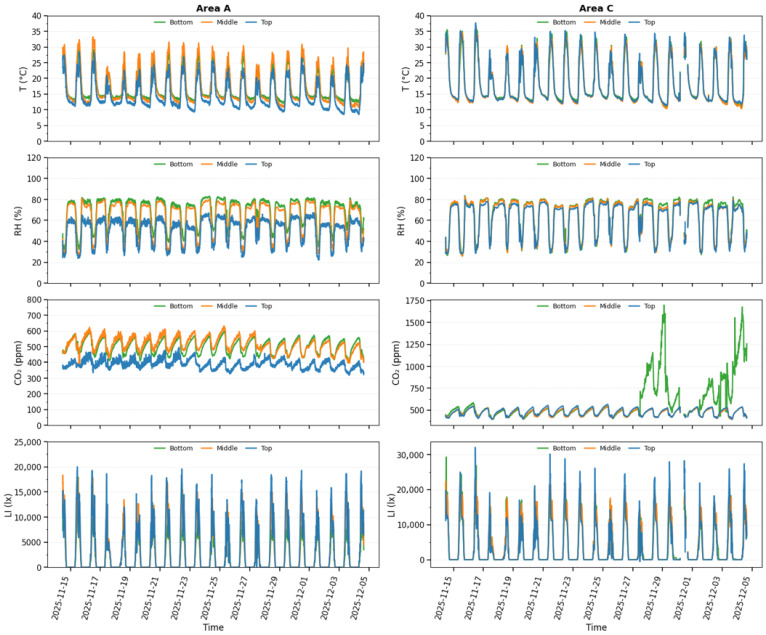
Temporal variation of temperature, relative humidity, CO_2_ concentration, and light intensity across vertical layers in greenhouse Section A and Section C.

Figure 12 shows clear diurnal patterns and vertical stratification of temperature (T), relative humidity (RH), CO_2_ concentration, and light intensity (LI) across Sections A and C. Daytime conditions exhibited higher temperature and light intensity at the top layer, while relative humidity was consistently higher near the bottom layer, reflecting crop transpiration and vertical airflow effects. CO_2_ concentration remained relatively stable in Section A but showed episodic accumulation at the bottom layer in Section C, indicating localized ventilation limitations.

**Figure 13:**
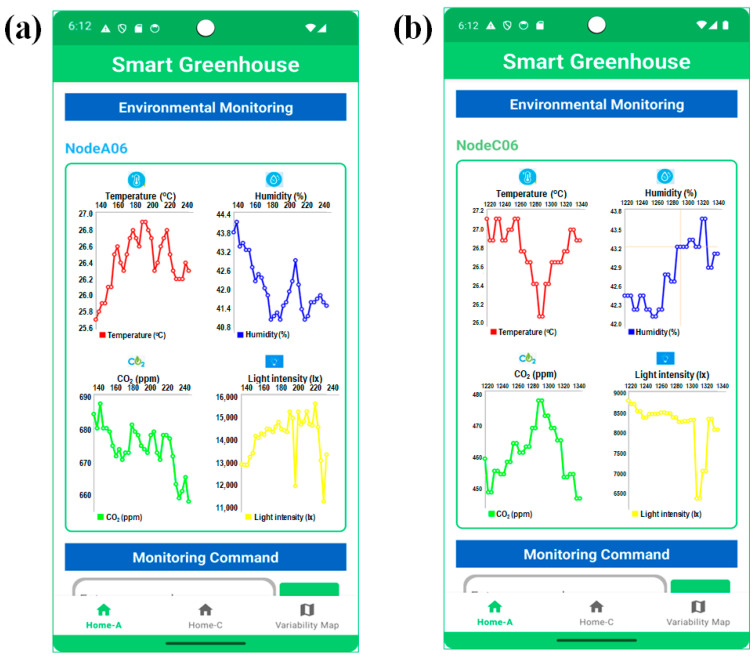
Smartphone application visualization of vertically resolved environmental conditions for representative nodes: (**a**) node A06 in Section A, and (**b**) node C06 in Section C. The x-axis represents consecutive samples at a fixed sampling interval of 1 min.

These vertically resolved environmental patterns were directly reflected in the real-time smartphone application through node-level visualization of temperature, humidity, CO_2_ concentration, and light intensity. Area A in Figure 12 presents the environmental time series data for Node A06 in greenhouse Section A, while Area C in Figure 12 illustrates the corresponding measurements for Node C06 in greenhouse Section C, highlighting clear vertical and temporal variability across the monitored layers. The smartphone application provided an intuitive, responsive monitoring interface, enabling users to identify vertical stratification and short-term temporal fluctuations in environmental conditions in real-time. The consistency between the time series analysis and smartphone visualization confirms the effectiveness of the proposed monitoring framework in capturing environmental heterogeneity within the smart greenhouse and supporting real-time awareness, as summarized in Figure 13.

### 3.2. Spatial Variability for Real-Time Remote Monitoring via Smartphone Applications

**Figure 14:**
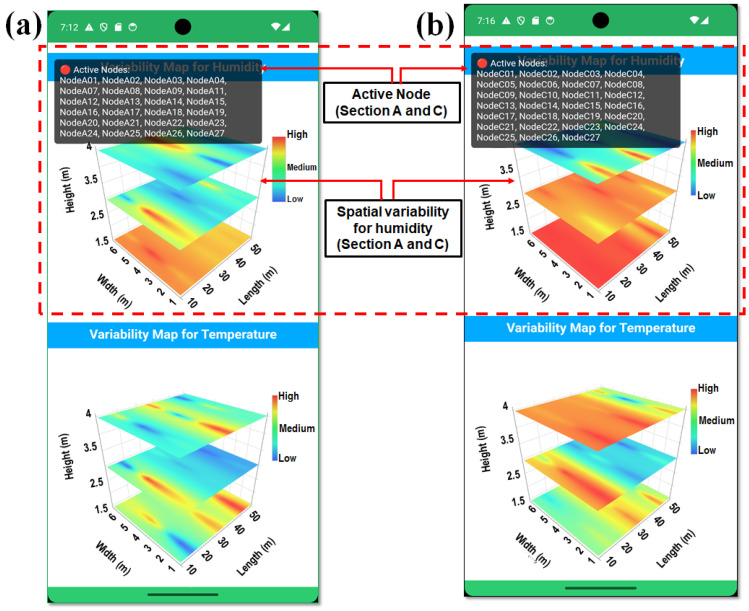
Real-time spatial variability visualization of humidity using a smartphone application for (**a**) Section A, and (**b**) Section C, illustrating vertically resolved humidity patterns based on active sensor node data (red dotted block).

The smartphone application visualized the spatial variability in humidity across the bottom, middle, and top layers in Sections A and C of the smart greenhouse. At 7:12 PM, humidity in Section A varied across layers, with the bottom layer having the highest levels (44.2–58.2%) due to crop transpiration and limited air circulation, while the middle layer (33.4–57.8%) showed more variability. The top layer had lower humidity (38.2–43.5%) due to roof-level ventilation. In Section C, the bottom layer had higher humidity (48.0–57.0%), while the middle layer showed more uniform moisture levels (47.5–54.6%), and the top layer had more stable humidity (40.0–43.5%), indicating better moisture regulation than in Section A, as shown in Figure 14. These findings, displayed using 3D interpolation and real-time data visualization, provide a detailed view of the smart greenhouse environmental conditions and highlight the effectiveness of the smartphone application for monitoring and optimizing environmental conditions. 

**Figure 15:**
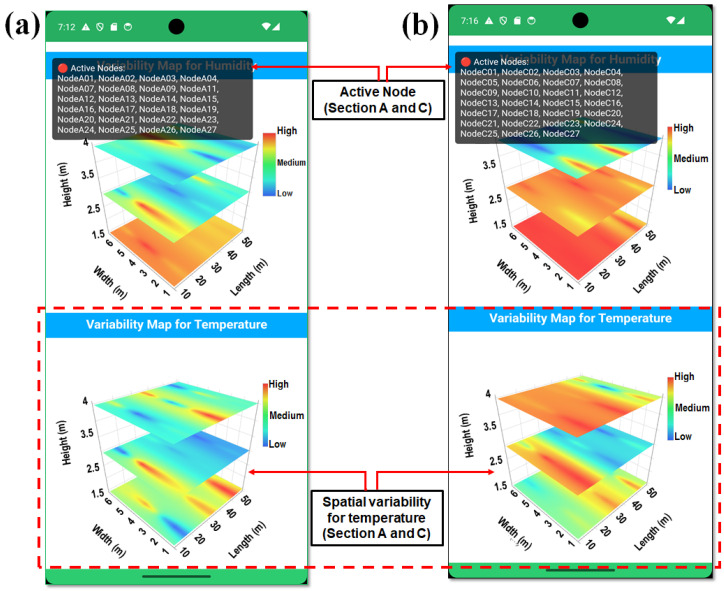
Real-time spatial variability visualization of temperature using a smartphone application for (**a**) Section A and (**b**) Section C, illustrating vertically resolved temperature patterns based on active sensor node data (red dotted block).

The spatial variability analysis for temperature in Sections A and C was conducted using real-time data from the sensing nodes, which were visualized via a smartphone application. In Section A, the bottom layer (nodes 1–9) showed stable temperatures (21.0–24.6 °C) due to moderate crop transpiration and shading. The middle layer (nodes 10–18) exhibited greater temperature fluctuations (27.0–33.2 °C), influenced by airflow variations. The top layer (nodes 19–27) recorded the highest temperatures (28.0–33.3 °C), due to increased solar exposure and heat transfer from the lower layers. Similarly, in Section C, the bottom layer showed temperatures ranging from 27.2 °C to 29.5 °C, with a cooling effect from higher humidity. The middle layer experienced a broader range (28.8–32.0 °C), while the top layer (29.8–33.5 °C) exhibited a more consistent temperature, thanks to better temperature management. These findings, displayed in Figure 15, show a clear vertical temperature gradient within the smart greenhouse, with the bottom layers cooler due to transpiration and shading, and the top layers warmer due to solar radiation and upward heat convection.

**Figure 16:**
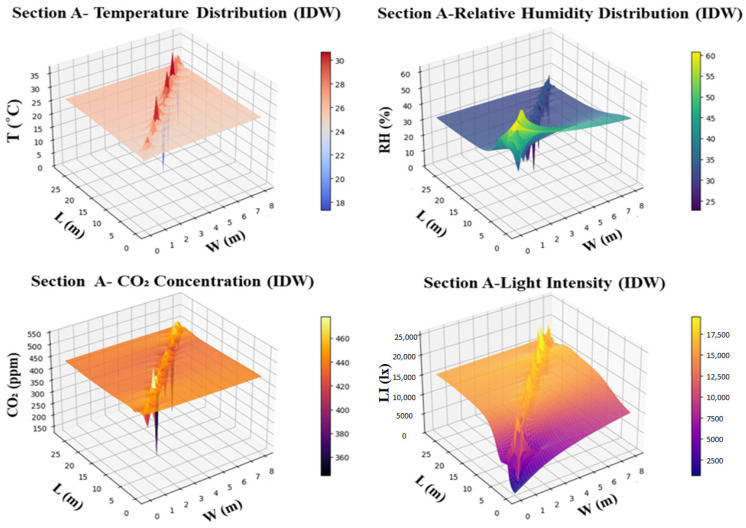
Spatial distribution of environmental parameters in smart greenhouse Section A using IDW interpolation.

In Section A of the smart greenhouse, environmental distribution was mapped using IDW interpolation, providing insights into the spatial variability of key parameters. Temperature showed a significant gradient, with values reaching 30 °C at the center and dropping to 18 °C at the edges, indicating heat buildup in the center of the smart greenhouse. Humidity levels also displayed variability, ranging from 55% at the edges to 25% at the center, suggesting a need for improved humidity control, especially near the plant canopy. CO_2_ concentration remained within a range of 360–460 ppm, though levels were slightly higher at the center, which could be attributed to localized plant respiration or reduced airflow. Light intensity exhibited the most pronounced variation, with 17,500 lx recorded at the center and only 2500 lx at the edges, indicating uneven light distribution across the section, as illustrated in Figure 16. This analysis underscores the need for targeted interventions, such as improved ventilation, humidity regulation, and better light distribution, to optimize the microclimate conditions for plant growth within Section A of the smart greenhouse. 

**Figure 17:**
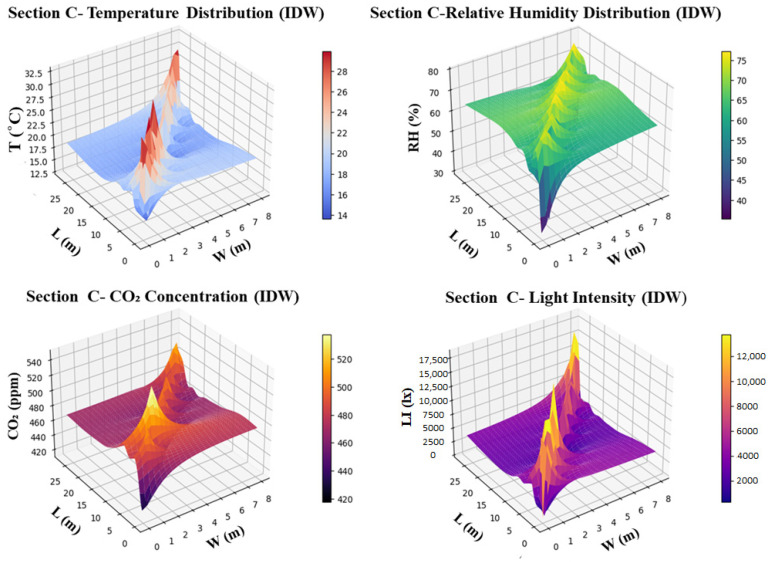
Spatial distribution of environmental parameters in smart greenhouse Section C using IDW interpolation.

In Section C of the smart greenhouse, the environmental distribution was analyzed using IDW interpolation to evaluate the spatial variability of critical parameters. Temperature measurements revealed a significant gradient, with a high of 32.5 °C at the center and a low of 17.5 °C at the edges, highlighting the buildup of heat at the center and indicating the need for improved heat distribution across the greenhouse. Humidity levels were higher in the center (70%) and lower at the edges (40%), suggesting that moisture control is required, particularly in the central region, to maintain optimal conditions for plant growth. CO_2_ concentrations ranged from 420 to 520 ppm, with slightly higher levels at the center, pointing to the need for CO_2_ distribution optimization to ensure uniform air quality throughout the greenhouse. Light intensity varied significantly, with the center receiving 17,500 lx and the edges only 2500 lx, emphasizing the need for better light management strategies to ensure uniform illumination for plant growth, as shown in Figure 17. Overall, the findings for Section C indicate areas that require targeted interventions, including heat, moisture, CO_2_, and light distribution improvements, to enhance environmental control and optimize plant growth conditions. 

### 3.3. Abnormal Signal Detection Notifications for Real-Time Remote Monitoring

**Figure 18:**
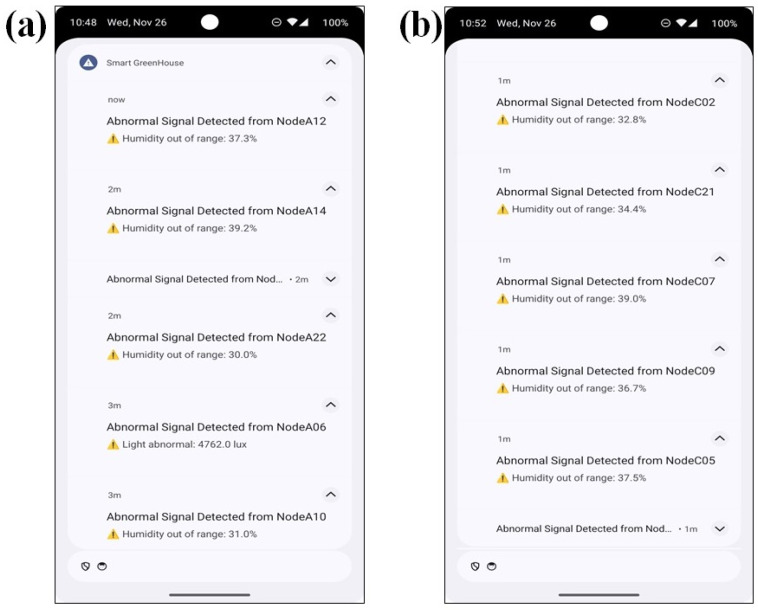
Abnormal signal detected visualization in smartphone application for real-time remote monitoring, (**a**) for Section A and (**b**) for Section C.

During the monitoring period, the smartphone application successfully identified abnormal environmental signals in both Section A and Section C, as shown in Figure 18. The abnormal notifications were generated when measured temperature, humidity, CO_2_, light intensity, and power consumption values exceeded predefined operational thresholds, demonstrating the system capability to detect node-level deviations in real-time monitoring in a smart greenhouse. These results demonstrate the system capability to detect node-level deviations in real-time within a commercial smart greenhouse environment.

In Section A, abnormal events were detected across multiple environmental and actuator nodes, indicating pronounced spatial variability in environmental conditions and actuator status. Nodes such as node A10, node A12, node A14, and node A22 showed significant variations in environmental parameters, with humidity levels fluctuating outside the expected range of 37.3% and 39.2%, respectively. These excursions suggest localized instability in soil moisture rather than uniform section-wide fluctuations. Node A06 recorded an abnormal light intensity of 4762.0 lx, which may reflect excessive localized illumination or sensor-specific inconsistencies. In addition, it elevated CO_2_ concentrations ranging from 780 to 790 ppm were observed at nodes A06 and A09, respectively, exceeding ambient baseline levels and indicating localized gas accumulation. The repeated detection of abnormal signals at these nodes confirmed that the monitoring framework can capture persistent, node-specific anomalies rather than isolated measurement noise.

In Section C, abnormal signals were also identified, though with a lower frequency than in Section A. Nodes C02, C05, C07, C09, and C21 exhibited humidity values outside the expected operational range, with measured levels between 36.7% and 39.0%, indicating localized dry-zone formation. Temperature anomalies were detected at nodes C05, C07, where values dropped to 12–14 °C, suggesting insufficient thermal regulation or airflow limitations in those locations. Elevated CO_2_ concentration of approximately 1200 ppm was recorded at node C02, indicating localized accumulation likely associated with limited ventilation. An abnormal light intensity of 4438 lx was observed at node C27, reflecting spatial non-uniformity in solar radiation distribution.

A quantitative summary of abnormal signal detections recorded over a 24 h period is presented in Table 5, which reports the affected nodes, abnormal parameter ranges, and the number of occurrences for each environmental variable. The results indicate that abnormal events were not uniformly distributed across the greenhouse, highlighting strong spatial dependence and localized environmental effects. These findings underscore the importance of continuous sensor calibration, systematic data validation, and spatially aware monitoring strategies to enhance data reliability and operational robustness in smart greenhouse management.

Therefore, the results confirm that the proposed real-time monitoring affects the detection and reports abnormal environmental and actuator conditions at the node level. The observed abnormal patterns indicate the importance of continuous sensor calibration, systematic data validation, and further investigation of localized environmental influences to improve data reliability and operational robustness in smart greenhouse management.

### 3.4. Smartphone Application Energy Consumption for Real-Time Remote Monitoring

**Figure 19:**
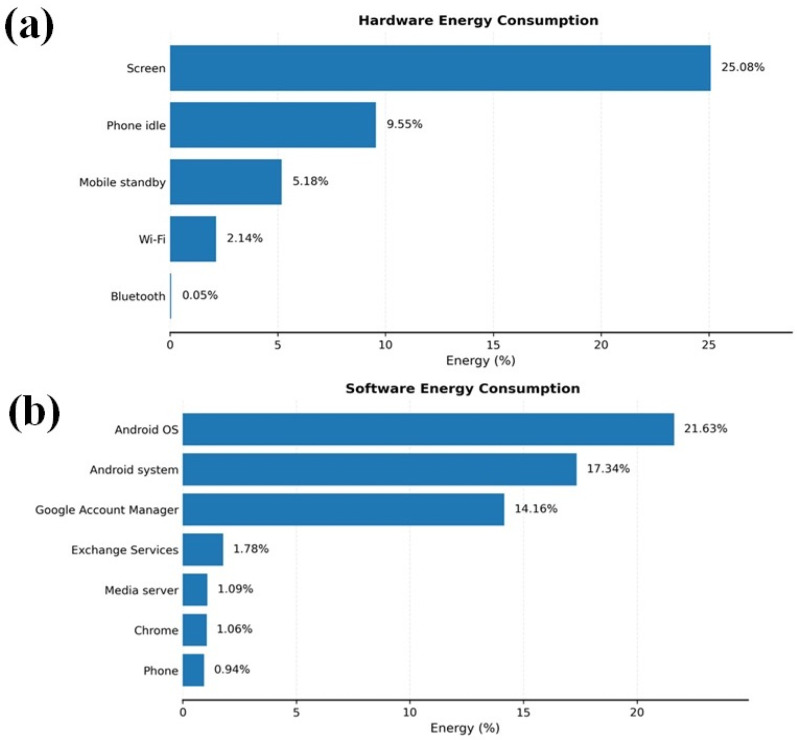
Energy consumption of (**a**) hardware and (**b**) software during the remote monitoring in a smart greenhouse via smartphone application.

The energy consumption of both the hardware and software components of the smartphone application was used for real-time environmental monitoring in the smart greenhouse. In the experiment conducted on a Samsung Galaxy A13, during the data collection and visualization of incoming data, the screen (25.08%) and communication modules (Wi-Fi, 2.14%) were the main hardware contributors to energy usage. On the software side, the Android system (17.34%) and Android OS (21.63%) were the primary energy consumers. The battery power and CPU load patterns indicated higher energy used during data updates and visualizations, as shown in Figure 19.

**Figure 20:**
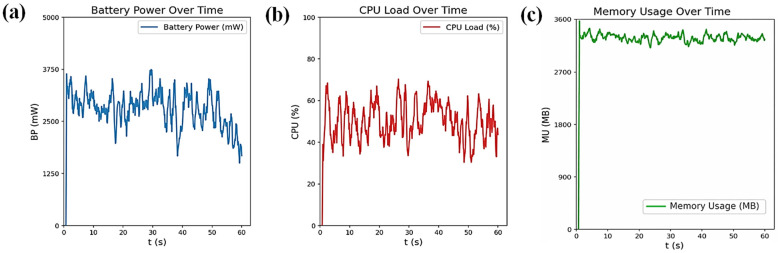
Battery (**a**), CPU (**b**), and memory utilization (**c**) during real-time remote monitoring in a smart greenhouse via smartphone application.

Profiling data communications of smartphone applications, as shown in Figure 20, was determined based on battery power, CPU load, and memory usage over time during real-time environmental monitoring in the smart greenhouse. Energy profiling was conducted as a feasibility assessment rather than an optimization study. Potential improvements, including adaptive sampling and reduced visualization frequency, were identified as future research directions. Spikes in battery power and CPU load occurred when data were processed or transmitted, particularly around t = 0 s and t = 60 s, reflecting moments of high computational demand. Memory usage remained relatively stable with minor fluctuations, indicating consistent data storage and management. These patterns highlight that energy consumption was driven by data-intensive tasks like transmission and processing, suggesting opportunities for optimization to improve energy efficiency while maintaining real-time monitoring. Based on measured power consumption (peak ≈ 3.5 W, lower during steady monitoring), the smartphone application was operationally feasible for several hours of continuous use, which aligns with typical daily greenhouse inspection and supervision cycles. 

These findings point to potential optimization strategies that could improve energy efficiency without compromising smartphone application functionality. Adjusting the data update frequencies, adopting low-power communication protocols, and reducing background processes could help decrease energy consumption. By implementing these strategies, the smart greenhouse monitoring system could operate more sustainably while maintaining the necessary real-time monitoring capabilities.

## 4. Discussion

This study developed and experimentally validated an Android-based smartphone edge monitoring system integrated with a multi-layer wireless sensor and actuator network for real-time environmental sensing, spatial visualization, and abnormal signal detection in a commercial smart greenhouse. The proposed application extends beyond conventional greenhouse dashboards by incorporating vertically stratified spatial mapping, deterministic anomaly detection, actuator power diagnostics, and on-device edge processing within a unified supervisory framework. Therefore, it lies not merely in interface development, but in demonstrating that dense multi-layer sensing and actuator networks can be reliably supervised in real-time through a smartphone-based edge architecture under sustained commercial operating conditions. Limited to a single commercial greenhouse with a crop (strawberry), and over a two-week late-autumn monitoring period. Seasonal effects, cross-site variability, and different greenhouse structures were not evaluated and will be addressed in future multi-site studies. The proposed system addressed several limitations of conventional greenhouse monitoring platforms, particularly the lack of vertical sensing resolution, limited spatial mapping capability, delayed cloud-based processing, and insufficient anomaly detection functionality [18,20,35,38]. By combining LoRaWAN-based data transmission, smartphone visualization, and node-level anomaly detection, the system provides a more responsive and spatially aware monitoring framework for greenhouse management. Commercial greenhouse environments present unique wireless deployment conditions that extend beyond controlled laboratory settings. High relative humidity and condensation can increase signal attenuation, particularly within dense canopy zones where moisture accumulation alters local propagation characteristics. Metallic structural components, such as steel frames, rails, heating pipes, and actuator housings, introduce multipath fading and localized shadowing, while the frequent switching of motors, fans, and compressors generates transient electromagnetic interference. These factors introduce variability in packet reception and link stability under operational conditions.

Wireless communication reliability is influenced by additional constraints reported in industrial and multi-hop wireless sensor network literature. These include packet collisions under dense node deployment, gateway congestion during synchronized uplink bursts, duty-cycle limitations in low-power wide-area networks, antenna polarization mismatch, progressive canopy growth altering propagation paths, and clock drift in asynchronous scheduling. Short-duration interference generated by high-current actuator switching may further degrade temporary link stability.

To mitigate such challenges, prior studies have proposed time-synchronized slot scheduling, adaptive channel and spreading factor allocation, redundancy-based retransmission mechanisms, forward error correction, congestion-aware routing, and resource-optimized multi-hop transmission strategies to reduce latency and packet loss under high-traffic conditions. These protocol-level strategies are compatible with the LoRaWAN–MQTT architecture adopted in this study and could enhance robustness in larger-scale deployments. However, the objective of the present work was not to redesign lower-layer communication protocols but to validate end-to-end system stability, sustained data synchronization, and supervisory monitoring performance under realistic commercial greenhouse workloads. Communication reliability was therefore evaluated at the system level rather than at the physical or MAC layer.

During the deployment of 54 sensing nodes and 12 actuator nodes at a 1-min sampling interval (over 1.08 million environmental data points), continuous data synchronization was maintained without sustained packet loss or system-level interruption. Temporary anomalies were captured and buffered at the edge layer when required, indicating operational robustness under realistic greenhouse conditions. Communication reliability, latency behavior, and smartphone energy profiling are therefore reported as feasibility indicators under real deployment workloads rather than as protocol-level innovations.

Extensive research has improved communication reliability in IoT networks through multi-hop routing, congestion-aware scheduling, adaptive spreading factor allocation, and forward error correction. These protocol-level strategies are essential for wide-area or infrastructure-scale deployments. However, the objective of the present study was not to redesign lower-layer communication mechanisms, but to validate end-to-end supervisory stability under greenhouse-scale operating conditions. Given the confined spatial dimensions of the experimental site and stable link quality achieved through adaptive data rate (ADR), single-hop LoRaWAN communication provided reliable performance without additional routing complexity. The contribution, therefore, lies in system-level integration and supervisory validation rather than protocol-layer innovation [10,11,12,13,14,40].

The experimental results demonstrated distinct vertical stratification in temperature, relative humidity, CO_2_ concentration, and light intensity across the bottom, middle, and top greenhouse layers. Daytime temperature differences of up to 5–7 °C between canopy and roof levels, together with localized CO_2_ accumulation exceeding 1500 ppm, indicate strong airflow and limited vertical mixing under high solar radiation conditions. Such three-dimensional microclimate variability is difficult to capture using single-layer or horizontally distributed sensor deployments commonly reported in earlier studies [15,16,35,36]. By deploying 27 wireless sensor nodes per section and organizing them into three vertical layers, the proposed system achieved high spatial resolution and enabled the accurate characterization of thermal stratification, inverse humidity gradients driven by crop transpiration, CO_2_ buildup in poorly ventilated canopy zones, and light attenuation from the roof to lower plant layers. This multi-layer sensing architecture provides a more representative assessment of greenhouse microclimate dynamics than traditional single-point sensing approaches [9,35].

The smartphone application enabled the real-time visualization of environmental parameters using node-level data streaming and IDW-based spatial interpolation. The results revealed strong spatial heterogeneity, with temperature ranging from 17.5 °C to 32.5 °C, humidity varying between 25% and 70%, and light intensity differing from 200 lx to 17,500 lx within the same greenhouse sections. Such variability confirms that greenhouse environments are highly non-uniform, even over short spatial distances. Previous studies often relied on fixed computer dashboards or cloud-based visualization interfaces, which limited real-time field usability and spatial awareness [21,25,36]. In contrast, the smartphone–edge interface in this study provided low-latency, mobile visualization that allowed operators to identify localized microclimate anomalies and implement targeted control actions directly in the greenhouse. This capability is particularly important for addressing uneven heat distribution, moisture accumulation, and light penetration, which are known to influence crop growth uniformity and physiological stress [7,24].

A major contribution of this work is the implementation of real-time, node-level anomaly detection directly on the smartphone platform. The system detected abnormal environmental conditions such as extreme temperature values up to 45.5 °C, low humidity zones between 36.7% and 39.0%, elevated CO_2_ concentrations around 1200 ppm, and abnormal light intensities exceeding 4400 lx. Unlike cloud-based anomaly detection systems that suffer from communication latency and limited spatial resolution [38], the proposed edge-based approach enabled the rapid detection of localized deviations. The repeated detection of anomalies at specific nodes confirms that the system identified persistent environmental or actuator-related issues rather than transient sensor noise, thereby improving fault localization and operational reliability [20,27]. Earlier greenhouse monitoring platforms often lacked integrated anomaly detection or relied on simple threshold-based alerts without spatial context [35,37]. The integration of real-time sensing, spatial mapping, and abnormality detection within a unified smartphone interface therefore, represents a significant improvement in system intelligence and usability.

The system used a LoRaWAN-based communication architecture to enable long-range, low-power data transmission from distributed sensor nodes to the gateway and cloud platform. LPWAN technologies such as LoRaWAN are well-suited for greenhouse environments due to their robustness against signal attenuation caused by humidity, vegetation, and metallic structures [10,11,12,13,14,40]. The stable data flow observed during the experiments confirms its suitability for multi-node greenhouse deployments. Compared with ZigBee or Wi-Fi, LoRaWAN provides improved coverage and energy efficiency, supporting dense sensor networks for capturing spatial and vertical microclimate variability [12,13].

Energy profiling showed that the smartphone displays and communication modules were the main hardware power consumers, while Android OS and system services dominated software energy usage. Battery and CPU load peaks occurred during intensive data transmission and real-time visualization, consistent with previous Android-based IoT applications [42]. Compared to cloud-centered architectures [25,26], the smartphone–edge approach reduces communication overhead while maintaining real-time responsiveness. Further optimization through adaptive sampling and lightweight data processing could improve energy efficiency.

Most existing greenhouse monitoring systems rely on planar sensor deployment and cloud-based visualization, lacking vertical sensing, spatial interpolation, node-level anomaly detection, and on-device processing [35,36,37,43]. Although some studies have introduced cloud-based anomaly detection [38] or actuator monitoring [41,44,45], these features are often implemented as isolated modules. In contrast, the proposed system integrates multi-layer sensing, LoRaWAN communication, smartphone–edge visualization, real-time anomaly detection, and actuator monitoring into a unified framework, providing higher spatial fidelity and faster response.

Three-dimensional microclimate monitoring enables more precise environmental control, including targeted ventilation, localized humidity regulation, optimized CO_2_ distribution, and improved light management. These strategies enhance crop stability, reduce resource waste, and support proactive greenhouse management through real-time alerts [4,5,24]. Future improvements may include predictive environmental models, additional crop and soil sensors, adaptive control algorithms, and edge-based machine learning for advanced anomaly classification [39]. To systematically position the proposed platform relative to existing studies, Table 6 compares the sensing configuration, visualization capability, anomaly detection location, interface type, and validation environment across representative smart greenhouse monitoring systems.

This study presents a unique combination of multi-layer sensor networks, real-time spatial and vertical variability mapping, and on-device anomaly detection, resulting in a more complete and efficient solution for smart greenhouse management. This study’s capacity to analyze vertical environmental fluctuations and detect localized abnormalities in real-time outperforms earlier sensor-based systems.

## 5. Conclusions

This study developed and validated an Android-based smartphone–edge monitoring system integrated with a multi-layer wireless sensor and actuator network for real-time environmental monitoring, spatial visualization, and abnormal signal detection in a commercial smart greenhouse. The system addressed key limitations of conventional greenhouse monitoring platforms by enabling three-dimensional sensing, node-level anomaly detection, and low-latency smartphone-based visualization.

Experimental results demonstrated pronounced vertical and spatial variability in greenhouse microclimate conditions. Daytime temperature differences of up to 5–7 °C were observed between the bottom and top layers, while relative humidity varied from 25% to 70% within the same greenhouse sections. Localized CO_2_ accumulation exceeding 1500 ppm was detected in poorly ventilated canopy zones, and light intensity showed strong spatial non-uniformity, ranging from 2500 lx at the edges to 17,500 lx near the center. These findings confirm the importance of multi-layer sensor deployment for accurately capturing three-dimensional microclimate dynamics.

The smartphone application successfully visualized real-time environmental data using node-level streaming and spatial interpolation, enabling operators to identify localized environmental heterogeneity. On-device anomaly detection identified abnormal conditions such as extreme temperature (45.5 °C), low humidity zones (36.7–39.0%), elevated CO_2_ levels (~1200 ppm), and irregular light intensity (~4400–4700 lx). This edge-based approach reduced detection latency compared to cloud-centered architectures and improved fault localization at the node level. The LoRaWAN-based communication infrastructure provided reliable, long-range, and energy-efficient data transmission, supporting scalable multi-node deployments under greenhouse conditions characterized by high humidity and structural signal interference. Energy profiling showed that the smartphone displays (25.08%) and communication modules (2.14%) dominated hardware power consumption, while Android OS and system services accounted for approximately 39% of software energy usage.

Overall, the proposed system offers a practical and scalable solution for smart greenhouse management by integrating multi-layer sensing, real-time spatial visualization, anomaly detection, and mobile user interaction into a unified platform. Future work will focus on incorporating predictive environmental models, additional crop and soil sensors, adaptive control algorithms, and edge-based machine learning to enable more autonomous and proactive greenhouse climate management.

## Figures and Tables

**Table 1:** Summarizes the abnormality classification framework implemented in the mobile-edge monitoring architecture.

Class	Domain	Detection Condition	Parameterization	Temporal Rule	Interpretation
E1	Environmental Range	x_p,i,t_ → [x_p,min_, x_p,max_]	18 °C ≤ T ≤ 28 °C; 50% ≤ RH ≤ 85%; 1250 ppm ≤ CO_2_ ≤ 1500 ppm; 5000 ≤ LI ≤ 40,000 lux	Instantaneous	Unsafe environmental condition
E2	Environmental Statistical	$|x_{i,t}-\mu_{i,w}|\;>3\sigma_{i,w}$	Rolling window (*w* = 15)	≥3 consecutive samples	Environmental disturbance or sensor anomaly
A1	Actuator Power	$c_{i,t}-c_{i}^{expected}|\;>\delta_{c}\;c_{i}^{expected}$	$\mathrm{Power}\;\mathrm{deviation}\;\mathrm{threshold}\;\delta_{c}=0.20$	Single or sustained deviation	Actuator fault
A2	Actuator Functional	$u_{i,t}=1,\;|\;|x_{i,t+\tau }-x_{i,t}|$	$\mathrm{Response}\;\mathrm{timeout}\;T_{response}=2$ intervals	After timeout	Ineffective actuator response
C1	Communication	$t_{current}-t_{i,last}>T_{timeout}$	$\mathrm{Communication}\;\mathrm{timeout}\;T_{timeout}=3$ intervals	Immediate	Node disconnection

**Table 2:** Specifications of the environmental sensors used in the study.

Sensor Type	Temperature and Humidity	Carbon Dioxide	Light Intensity
Image	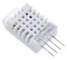	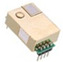	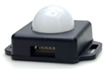
Detection range	Temp: −40~80 °C Hum: 0~100%	0~5000 ppm	1~65,535 lux
Accuracy	Temp: ±0.5 °C; Hum: ±2%	±50 ppm	-

**Table 3:** Specifications of the actuator sensors used in the study.

Sensor Type	Current Sensor
Image	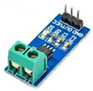
Detection range	±30 A
Accuracy	±1.5%

**Table 4:** Per-node statistical summary of environmental parameters across Sections A and C (statistics computed at node level over the two-week monitoring period).

Variable	Section	Mean of Node Means	SD Across Nodes	Median IQR (Q3–Q1)
**Temperature (°C)**	A	26.63	2.14	3.82
	C	32.59	1.37	2.11
**Relative** **humidity (%)**	A	30.53	2.68	4.26
	C	30.01	2.11	3.74
**CO_2_ (ppm)**	A	431.64	38.92	64.8
	C	423.92	9.84	18.3
**Light intensity (lx)**	A	11,196.32	1942.5	3280
	C	16,896.34	2684.1	4920

**Table 5:** Nodes with abnormal detection in the smart greenhouse.

Section	Parameters	Number of Affected Nodes	Nodes with Abnormal Detection	Abnormal Range
**A**	Temperature (°C)	1	A14	45.5 °C
	Relative humidity (%)	3	A10, A14, A22	37.3~39.2%
	CO_2_ (ppm)	2	A06, A09	780~920 ppm
	Light intensity (lx)	1	A06	4762.0 lx
**C**	Temperature (°C)	2	C05, C07	12 °C~14 °C
	Relative humidity (%)	5	C02, C05, C07, C09, C21	36.7~39.0%
	CO_2_ ppm	1	C02	1200 ppm
	Light intensity (lx)	1	C27	4438.0 lx

**Table 6:** Comparison of the proposed Android-based smartphone application for smart greenhouse monitoring with related studies.

Aspect	Related Studies (Typical Approaches)	Proposed System (This Study)
Sensing configuration	Single point or limited nodes, single height [20,35]	Multi-layer (bottom, middle, and top) sensing with 54 nodes
Spatial visualization	Time series plot only, no spatial mapping [36]	Real-time 3D spatial variability maps on a smartphone
User interface	Web dashboards or PC based monitoring [37]	Full smartphone native interface
Anomaly detection	Cloud-side or control-based processing [38]	On-device (mobile-edge) anomaly detection
Detection latency	Dependent on cloud round-trip delays [39]	Low-latency local processing
Communication technology	Wi-Fi/ZigBee, limited range or higher power use [40]	LoRaWAN + MQTT (long-range, low power)
Actuator supervision	Basic control without diagnostic [41,44,45]	Integrated actuator status and power monitoring
App performance analysis	Rarely reported or omitted [42]	Battery, CPU, and memory profiling included
Validation environment	Laboratory or small-scale testbeds [43]	Commercial greenhouse deployment

## Data Availability

Data are contained within the article.
